# Effects of exercise mode and intensity on patient-reported outcomes in cancer survivors: a four-arm intervention trial

**DOI:** 10.1007/s00520-023-07757-9

**Published:** 2023-05-02

**Authors:** Fabian Pelzer, Kai Leisge, Kathrin Schlüter, Justine Schneider, Joachim Wiskemann, Friederike Rosenberger

**Affiliations:** 1 Working Group Exercise Oncology, Department of Medical Oncology, National Center for Tumor Diseases (NCT), Heidelberg University Hospital, Im Neuenheimer Feld 460, 69120 Heidelberg, Germany; 2 Institute of Sports and Sport Science, Heidelberg University, Heidelberg, Germany; 3 Division of Health Sciences, German University of Applied Sciences for Prevention and Health Management, Saarbruecken, Germany

**Keywords:** Fatigue, Quality of life, Aerobic training, Resistance training, Undulating, Polarized

## Abstract

**Purpose:**

The aim of this study was to compare the effects of different exercise modes (aerobic, resistance) and intensity prescriptions (standard, polarized, undulating) on patient-reported outcomes (PROs) in cancer survivors.

**Methods:**

107 breast or prostate cancer survivors (52% females, age 58 ± 10 years, 6–52 weeks after primary therapy) performed one out of four training programs, two sessions/week, over 12 weeks: work rate-matched vigorous intensity aerobic training (AT_Standard_, *n* = 28) and polarized intensity aerobic training (AT_Polarized_, *n* = 26) as well as volume-matched moderate intensity resistance training (RT_Standard_, *n* = 26) and daily undulating intensity resistance training (RT_Undulating_, *n* = 27). Health-related quality of life (HRQoL, EORTC-QLQ-C30) and cancer-related fatigue (CRF, MFI-20) were assessed at baseline, at the end of intervention and after a 12-week follow-up without further prescribed exercise.

**Results:**

Over the intervention period, HRQoL-function-scales of the EORTC-QLQ-C30 improved over time (*p* = .007), but no group*time interaction was observed (*p* = .185). Similarly, CRF values of the MFI-20 improved over time (*p* = .006), but no group*time interaction was observed (*p* = .663). When including the follow-up period and pooling the AT and the RT groups, HRQoL-function-scales developed differently between groups (*p* = .022) with further improvements in RT and a decline in AT. For CRF no significant interaction was found, but univariate analyses showed a non-significant trend of more sustainable effects in RT.

**Conclusions:**

AT and RT with different work rate-/volume-matched intensity prescriptions elicits positive effects on HRQoL and CRF, without one regimen being significantly superior to another over the intervention period. However, RT might result in more sustainable effects compared to AT over a follow-up period without any further exercise prescription.

**Clinical trial registration:**

The study was registered at clinicaltrials.gov (NCT02883699).

## Introduction

Health-related quality of life (HRQoL) and cancer-related fatigue (CRF) are highly relevant outcomes for cancer survivors. Their importance even rises in view of higher survival rates due to early detection and better treatment options [1]. Over the past decades, research has consistently demonstrated the positive effects of exercise on both endpoints. Today, the American Society of Clinical Oncology (ASCO) summarizes significant small effects of exercise on HRQoL and significant moderate effects of exercise on CRF during cancer treatment [2]. Furthermore, the Consensus Statement from International Multidisciplinary Roundtable recommends with strong evidence aerobic training, resistance training or a combination of both for cancer survivors [3]. However, there remains debate on the most effective exercise prescription.

The currently recommended frequency, intensity, time and type of exercise (FITT criteria) proved successful in previous studies with strong evidence for outcomes like HRQoL, CRF, anxiety and depressive symptoms [3]. However, these are mainly so-called first-generation studies that compared one exercise group to a control group. The lack of direct comparisons between multiple exercise groups limits knowledge on the optimal FITT criteria to address HRQoL and CRF.

Landmark second-generation studies comparing aerobic and resistance training or different intensity prescriptions include the Canadian START trial which compared aerobic vs. resistance training vs. usual care in breast cancer patients during chemotherapy [4]. It revealed no differences between aerobic and resistance training for HRQoL and CRF, with a trend in favor of the exercise groups compared to usual care. The subsequent CARE trial compared lower vs. higher dose of aerobic training vs. higher dose of combined aerobic and resistance training [5]. The higher dose aerobic training was borderline superior to the lower dose aerobic training in terms of CRF. Furthermore, the Swedish OptiTrain trial demonstrated that a combination of resistance and high-intensity interval aerobic training (HIIT) is superior to a combination of moderate intensity aerobic training and HIIT regarding CRF during chemotherapy [6]. None of the previous studies compared different exercise intensity prescriptions within one type of training and none was conducted in the post-treatment phase.

To fill this research gap, we conducted a four-arm exercise intervention trial with two aerobic training groups and two resistance training groups with different intensity prescriptions of matched work-rate or volume, respectively, in breast and prostate cancer survivors. The aim of this analysis was to compare the effects of the different exercise regimens on HRQoL and CRF which were assessed as secondary outcomes. The data will help finding the optimal dose prescription of the “exercise medicine” for cancer survivors which is considered one of the top research questions in exercise oncology [7].

## Materials and methods

### General design

We conducted a four-arm exercise intervention trial with two aerobic training (AT) groups and two resistance training (RT) groups (TOP-Study, clinicaltrials.gov: NCT02883699). The intervention lasted 12 weeks with two training sessions/week. Patients in the “standard aerobic training group” (AT_Standard_) performed vigorous-intensity continuous training, while patients in the “polarized aerobic training group” (AT_Polarized_) performed polarized training of matched total work, alternating between high-intensity interval training (HIIT) and moderate-intensity continuous training. Patients in the “standard resistance training group” (RT_Standard_) performed moderate-intensity resistance training, while patients in the “daily undulating resistance training group” (RT_Undulating_) varied between low-, moderate-, and high intensity resistance training of similar total weight. PRO’s were assessed as secondary outcomes of TOP-Study. Assessments were performed at baseline (t0), after 12 weeks of intervention (t1), and after 12 weeks of follow-up without any further prescribed exercise (t2). The study was in accordance with the Declaration of Helsinki and approved by the ethics committee of the Medical Faculty of Heidelberg (S-347/2016).

### Participants

Patients were recruited at a comprehensive cancer center and local oncologists as well as via a cancer registry and advertisement in self-help magazines. All participants met the following inclusion criteria: diagnosed with non-metastatic (M0) breast cancer or non-metastatic or metastatic prostate cancer (M0 or M1, except for bone or brain metastases, with PSA evidence of stable disease), 6 to 52 weeks after the end of primary therapy (i.e. surgery and/or radio therapy and/or chemotherapy), 18 to 75 years of age, and physically inactive (no regular aerobic or resistance training (> 1 session/week) since diagnosis or within the last 6 months). Exclusion criteria were: diagnosis with additional other cancer and severe comorbidities that precluded participation in exercise testing or training (acute infectious diseases, severe cardiac, respiratory, renal or neurological diseases). Current hormone therapy was allowed. Participants signed a written informed consent document before taking part in the study.

**Fig. 1:**
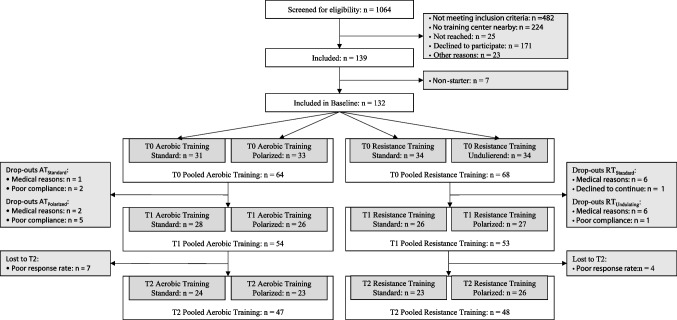
Participant flow chart

A participant flow chart is given in Fig. 1. Participants were allocated to AT or RT depending on available training machines at the study training facilities nearby patients’ homes. After baseline testing, they were randomized between AT_Standard_ and AT_Polarized_ or RT_Standard_ and RT_Undulating_ using a minimization procedure for the type of cancer (equivalent to sex), age, current hormone treatment and baseline fitness level, expressed as relative VO_2peak_ in the AT groups or relative maximal voluntary isometric contraction (MVIC) of the quadriceps in the RT groups. A total of 107 patients were finally analyzed over the intervention period (t0 to t1). Their characteristics are given in Table 1. Furthermore, sustainability of the intervention was analyzed including the follow-up (t0 to t2) in a total of 96 patients.

**Table 1:** Patients’ characteristics

	Training			
	AT_Standard_	AT_Polarized_	RT_Standard_	RT_Undulating_
Type of cancer, *n* (%)				
Breast cancer Prostate cancer	15 (53.6) 13 (46.4)	12 (46.2) 14 (53.8)	16 (61.5) 10 (38.5)	13 (48.1) 14 (51.9)
Age [years], mean ± SD	59 ± 10	60 ± 8	55 ± 11	61 ± 10
BMI [kg/m^2^], mean ± SD	27.47 ± 5.49	27.38 ± 5.07	26.90 ± 4.65	26.60 ± 4.07
Time since diagnosis [months], mean ± SD	13 ± 7	21 ± 19	27 ± 38	17 ± 15
Time since end of therapy [wk], mean ± SD	23 ± 14	25 ± 12	27 ± 20	28 ± 14
Tumor staging, *n* (%)				
0 I II III IV Unclear	0 (0) 10 (35.7) 11 (39.3) 6 (21.4) 0 (0) 1 (3.6)	0 (0) 13 (50) 4 (15.4) 7 (26.9) 0 (0) 2 (7.7)	0 (0) 12 (46.2) 6 (23.1) 7 (26.9) 1 (3.8) 0 (0)	0 (0) 13 (48.1) 11 (40.7) 2 (7.4) 0 (0) 1 (3.7)
Cancer treatment, *n* (%)				
Surgery alone Radio therapy alone Surgery + radio therapy Surgery + radio therapy + chemotherapy Additional hormone therapy Additional antibody therapy Unknown	6 (21.4) 1 (3.6) 10 (35.7) 9 (32.1) 15 (53.6) 5 (17.9) 2 (7.2)	2 (7.7) 4 (15.4) 14 (53.8) 4 (15.4) 15 (57.7) 2 (7.7) 2 (7.7)	4 (15.4) 1 (3.8) 9 (34.6) 11 (42.3) 15 (57.7) 5 (19.2) 1 (3.8)	7 (25.9) 4 (14.8) 9 (33.3) 6 (22.2) 13 (48.1) 2 (7.4) 1 (3.7)

No significant between group differences for type of cancer, age, BMI, time since diagnosis and time since end of therapy were found (BMI, body-mass-index)

### Outcome measures

HRQoL was assessed using the 30-item self-assessment questionnaire of the European Organisation for Research and Treatment of Cancer (EORTC-QLQ-C30). It consists of five functional scales (physical, role, emotional, cognitive, and social function), nine symptom scales (fatigue, pain, nausea/vomiting, dyspnoea, insomnia, appetite loss, constipation, diarrhoea and financial difficulties), as well as a general quality of life (QoL) scale (range: 0–100) [8, 9].

CRF was assessed using the multidimensional fatigue inventory (MFI-20). The Instrument consists of 20 items which represent five different dimensions of CRF (general fatigue, physical fatigue, mental fatigue, reduced activity, and reduced motivation; range: 4–20) [10].

### Exercise testing for training prescription

In the AT groups, a cardiopulmonary exercise test (CPET) on the cycle ergometer was performed to derive training prescriptions. In the RT groups, isometric and isokinetic strength tests on a stationary dynamometer and one-repetition maximum (1-RM) tests on training machines were performed.

### Training interventions

All training was performed indoors under gym-like conditions with close supervision by specialized exercise-therapists. The AT groups trained on cycle ergometers. AT_Standard_ performed two sessions/week, 30 min/session of vigorous-intensity continuous training at 97% individual-anaerobic-threshold (IAT, corresponding to 98 ± 18 W on average). AT_Polarized_ performed 1 session/week, 38 min/session of HIIT, starting with a 10 min warm-up at 70% HR_peak_, followed by 4 × 4 min intervals at 85–95% HR_peak_ (corresponding to 114 ± 29 W on average), interspersed with 3 min recovery at 70% HR_peak_ and finished with a 3 min cool-down at 70% HR_peak_. Furthermore, AT_Polarized_ performed 1 session/week of moderate-intensity continuous training at the LT (corresponding to 70 ± 18 W on average). The duration was chosen to be work rate-matched with AT_Standard_. Intensity was prescribed by heart rates and work rate was increased if the heart rate dropped below the target zone to maintain intensity over the course of the intervention. While a typical polarized intensity distribution for athletes includes 75–80% low intensity, 5% threshold intensity and 15–10% high intensity training, we adapted the principle of two clearly distinct intensity zones to untrained cancer patients who were capable of only two training sessions per week. 

Both RT groups performed two sessions/week of resistance training at six training machines for the major muscle groups. RT_Standard_ performed 3 sets at 67% 1RM. RT_Undulating_ varied between 2, 3, 4 or 5 sets, and 4 repetitions at 90% 1RM, 12 repetitions at 67% 1RM or 20 repetitions at 55% 1RM in recurrent order. Weight was increased in the subsequent session of the same type if the prescribed number of repetitions was achieved. The total weight moved was similar between RT groups.

### Statistical analyses

An appropriate sample size was estimated a priori based on the primary endpoint, not on the patient-reported outcomes reported here. For a minimum worthwhile difference in ΔVO_2peak_ between groups of 10%, a within-subject variation in VO_2peak_ of 5.6% [11], α = 0.05, and power = 80%, the estimation revealed *n* = 20 evaluable patients per group [12]. To account for potential drop-outs, a minimum of 30 patients per group were included.

Data were analyzed “intention to treat”. The statistical analysis was conducted using IBM SPSS Statistics 25 for Microsoft Windows (IBM Corp, Armonk, NY). To investigate changes in HRQoL and CRF over the intervention period, repeated measures multivariate analyses of variance (MANOVA) with the within-subject factor time (2, t0 – t1) and the between-subject factor group (4, AT_Standard_, AT_Polarized_, RT_Standard_, RT_Undulating_) were conducted for the EORTC-QLQ-C30 function scales, the EORTC-QLQ-C30 symptom scales, and the MFI-20. Due to the explorative nature of this study, univariate analyses for individual subscales were analysed even though no multivariate significance was present.

In a second step, sustainability of the training effects was investigated including the 12 weeks follow-up period. Both the AT groups and the RT groups were pooled to account for increasing heterogeneity (and decreasing sample-sizes) in this unstandardized period of the study. Pooling appeared appropriate because of the matched work rate or volume, respectively, within each type of training. A repeated measures MANOVA with the within subject factor time (3, t0—t2) and the between subject factor training (2, AT, RT) was used.

For all analyses, the Wilks-Lambda test statistic was used. The alpha level was set to 0.05. All data were checked for accuracy and therefore no extreme values were excluded from the data.

## Results

### Intervention period

**Fig. 2:**
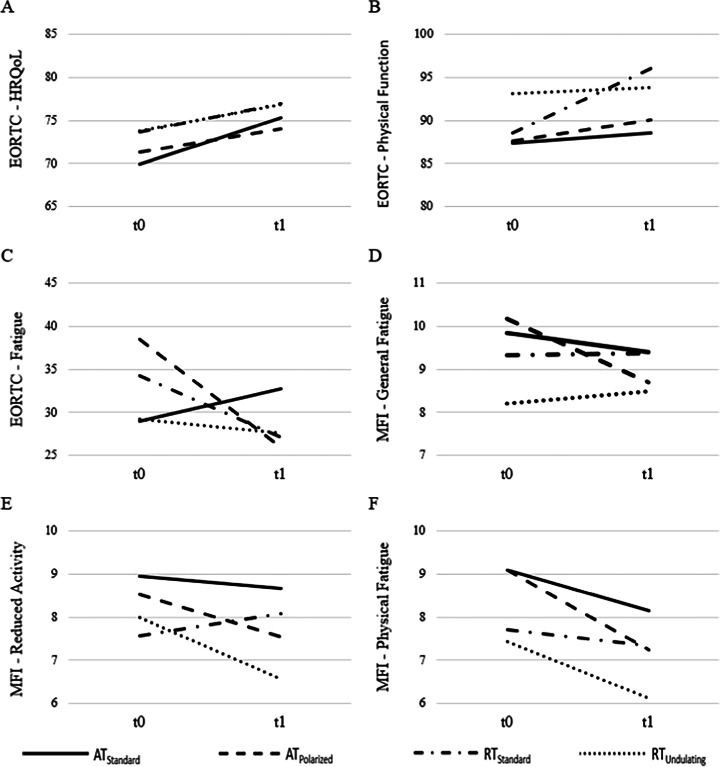
Changes in PRO’s during the intervention period. Mean changes over the intervention period for selected subscales of the EORTC-QLQ-C-30 and the MFI-20. Shown are EORTC-QLQ-C30 global health status (**A**) EORTC-QLQ-C30 physical function (**B**) EORTC-QLQ-C30 fatigue (**C**) (aerobic training standard group (AT_Standard_): *n* = 28; AT_Polarized_: *n* = 26; RT_Standard_: *n* = 26; RT_Undulating_: *n* = 27), MFI-20 general fatigue (**D**), MFI-20 physical fatigue (**E**) and MFI-20 reduced activity (**F**) (AT_Standard_: *n* = 25; AT_Polarized_: *n* = 23; RT_Standard_: *n* = 25; RT_Undulating_: *n* = 23) for the four training groups

Data of the intervention period are displayed in Fig. 2 for selected subscales and in Table 2 for every subscale of the EORTC-QLQ-C30 and the MFI-20. For the function scales of the EORTC-QLQ-C30, there was no significant group effect, *F*(18, 277.671) = 0.616, *p* = 0.886, η^2^ = 0.036, but values improved over time, *F*(6, 98) = 3.194, *p* = 0.007, η^2^ = 0.164. The analyses showed no differences in the development over time between the training groups, indicated by a non-significant interaction of time x group, *F*(18, 277.671) = 1.302, *p* = 0.185, η^2^ = 0.074.

**Table 2:** Means (*M*) and standard deviations (*SD*) for AT_Standard_, AT_Polarized_, RT_Standard_ and RT_Undulating_ of every subscale from EORTC QLQ-C30 and MFI-20 in the intervention period

	Prescriptions							
	AT_Standard_		AT_Polarized_		RT_Standard_		RT_Undulating_	
	t0	t1	t0	t1	t0	t1	t0	t1
EORTC Function								
Global Health Status								
*M* *SD*	69.94 16.56	75.29 18.07	71.47 15.30	73.71 13.88	73.39 15.98	77.24 16.92	73.76 14.56	76.84 19.92
Physical Function								
*M* *SD*	87.38 15.95	88.57 13.19	87.43 18.01	89.74 13.88	88.71 14.45	96.15 6.57	93.08 19.92	93.82 9.77
Role Function								
*M* *SD*	74.40 28.86	84.52 19.73	78.21 24.38	85.89 22.94	84.61 21.04	88.46 14.73	87.65 20.97	92.59 15.56
Emotional Function								
*M* *SD*	73.32 21.87	74.11 19.55	64.74 27.71	72.75 26.72	68.91 27.03	72.11 23.56	76.54 20.41	74.38 21.54
Cognitive Function								
*M* *SD*	75 22.91	77.97 20.31	78.21 22.49	80.76 20.38	80.12 24.5	83.97 22.35	83.33 23.11	85.8 24.33
Social Function								
*M* *SD*	76.78 23.71	83.33 21.27	77.56 23.54	76.28 27.15	78.84 23.83	88.46 19.3	83.33 20.67	88.88 17.29
EORTC Symptom								
Fatigue								
*M* *SD*	28.96 21.24	32.73 24.30	36.96 28.26	26.06 23.29	35.89 27.81	26.92 23.96	29.21 20.24	27.25 22.08
Pain								
*M* *SD*	19.64 22.71	20.83 22.96	17.94 27.97	20.51 25.95	15.38 17.58	18.58 21.25	17.90 26.92	17.90 22.61
Insomnia								
*M* *SD*	42.85 36.12	39.28 36.34	44.87 36.44	33.33 31.26	35.89 32.55	33.33 31.26	24.69 23.73	30.86 29.12
MFI								
General Fatigue								
*M* *SD*	9.84 3.65	9.40 3.50	9.87 4.36	8.83 3.56	9.64 4.29	9.24 4.15	8.22 2.99	8.48 3.28
Physical Fatigue								
*M* *SD*	9.08 3.45	8.16 3.24	9.00 3.75	7.39 2.93	7.84 3.63	7.20 3.41	7.43 2.96	6.13 2.39
Reduced Activity								
*M* *SD*	8.96 4.11	8.68 4.01	8.22 3.46	7.61 3.08	7.92 3.52	8.00 3.54	8.00 3.06	6.57 2.69
Reduced Motivation								
*M* *SD*	7.28 2.81	6.92 3.04	7.09 2.25	7.39 2.72	6.68 2.35	6.44 2.39	6.35 1.79	6.39 2.31
Mental Fatigue								
*M* *SD*	8.88 3.95	8.40 3.57	8.09 3.48	7.78 3.49	7.88 3.96	7.64 4.12	7.87 3.99	7.22 4.48

In the explorative univariate analyses significant changes over time were found for global health status (*p* = 0.021, η^2^ = 0.050, Fig. 2A), physical function (*p* = 0.009, η^2^ = 0.064, Fig. 2B), role function (*p* < 0.001, η^2^ = 0.119) and social function (*p* = 0.006, η^2^ = 0.071). All other univariate between-group and interaction effects failed to reach significance (*p* ≥ 0.130).

For the symptom scales of the EORTC-QLQ-C30, most of the symptoms (nausea/vomiting, dyspnoea, appetite loss, constipation, diarrhoea, financial problems) were not sufficiently present (median equal to 0) in the sample of this study. Therefore, only the symptom scales fatigue, pain, and insomnia were included in the analyses. The results of the MANOVA revealed no difference between the four training groups, *F*(9, 245.958) = 0.525, *p* = 0.856, η^2^ = 0.015, and no change over time, *F*(3,101) = 2.687, *p* = 0.050, η^2^ = 0.074. The development of the groups over time was not different, *F*(9, 245.958) = 1.913, *p* = 0.051, η^2^ = 0.052.

The results of the univariate analyses showed a significant change only in fatigue (*p* = 0.021, η^2^ = 0.051, Fig. 2C), but not in pain or insomnia (*p* ≥ 0.284). Furthermore, the development over time differed between groups for fatigue only (*p* = 0.025, η^2^ = 0.086), with an increase in AT_Standard_ compared to decreases in all other groups.

For the MFI-20, no differences between the training groups were found, *F*(15, 243.331) = 0.852, *p* = 0.618, η^2^ = 0.046, but fatigue values improved over time, *F*(5, 88) = 3.481, *p* = 0.006, η^2^ = 0.165. The analyses revealed no differences in the development over time between the training groups, indicated by a non-significant interaction of time x group, *F*(15, 243.331) = 0.813, *p* = 0.663, η^2^ = 0.044. Univariate analyses showed a significant change for the physical fatigue scale only (p < 0.001, η^2^ = 0.134, Fig. 2F).

### Follow-up analyses

**Fig. 3:**
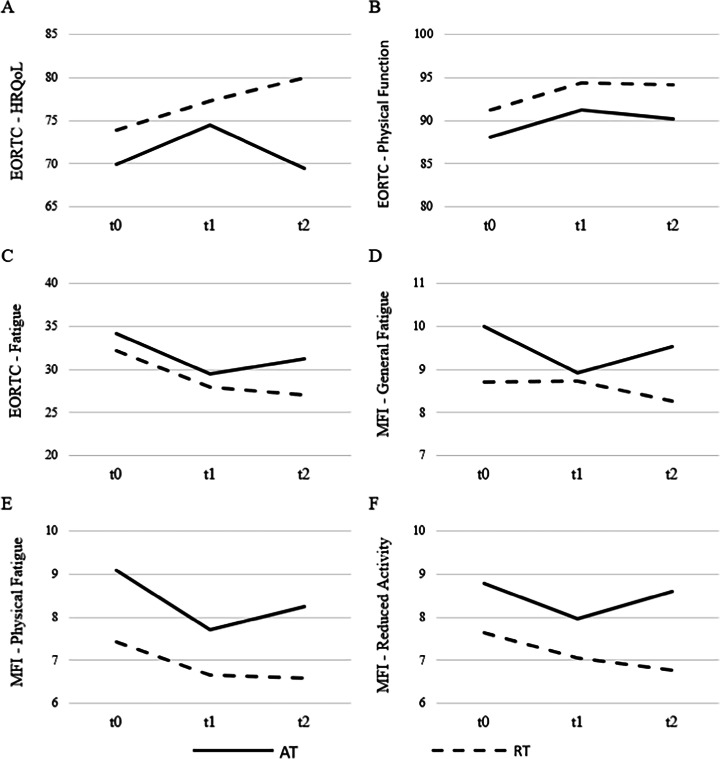
Changes in PRO’s during the follow-up period. Mean changes over the intervention and follow-up period for selected subscales of the EORTC-QLQ-C-30 and the MFI-20. Shown are EORTC-QLQ-C30 global health status (**A**) EORTC-QLQ-C30 physical function (**B**) EORTC-QLQ-C30 fatigue (**C**) (Aerobic Training (AT): *n* = 47; Resistance Training (RT): *n* = 49), MFI-20 general fatigue (**D**), MFI-20 physical fatigue (**E**) and MFI-20 reduced activity (**F**) (AT: *n* = 40; RT: *n* = 44) for the pooled training groups

Sustainability of the training effects (t0 – t2) pooled for the AT and RT groups is displayed in Fig. 3 for selected subscales and in Table 3 for every subscale. Regarding the function scales of the EORTC-QLQ-C30, there was no between group difference, *F*(6, 89) = 0.845, *p* = 0.539, η^2^ = 0.054, and no change over time, *F*(12, 83) = 1.720, *p* = 0.077, η^2^ = 0.199. However, the development of AT and RT was different over time with further improvements in RT and a decline in AT, *F*(12, 83) = 2.144, *p* = 0.022, η^2^ = 0.237.

**Table 3:** Means (*M*) and standard deviations (*SD*) for pooled AT- and RT-groups of every subscale from EORTC QLQ-C30 and MFI-20 during the follow-up period

	Prescription					
	Aerobic Training (pooled)			Resistance Training (pooled)		
	t0	t1	t2	t0	t1	t2
EORTC Function						
Global Health Status						
*M* *SD*	70.03 16.08	74.29 16.09	69.14 18.86	73.81 14.92	77.38 18.63	80.10 14.71
Physical Function						
*M* *SD*	87.94 15.70	91.06 11.48	89.92 13.46	91.29 12.33	94.55 8.57	94.28 12.69
Role Function						
*M* *SD*	75.887 26.87	84.75 21.37	80.49 27.21	86.39 21.15	90.13 15.55	87.75 19.47
Emotional Function						
*M* *SD*	68.26 25.03	71.63 23.92	68.97 27.56	73.97 24.27	74.48 22.39	79.08 20.27
Cognitive Function						
*M* *SD*	75.53 23.53	78.36 20.82	77.30 25.87	81.29 24.20	84.35 23.91	85.71 20.41
Social Function						
*M* *SD*	77.65 22.05	79.07 24.93	81.91 23.78	81.63 22.37	90.13 17.65	89.45 20.61
EORTC Symptom						
Fatigue						
*M* *SD*	33.21 24.74	29.66 24.08	31.21 27.08	33.11 24.10	27.89 23.58	27.21 24.95
Pain						
*M* *SD*	18.79 25.68	20.56 23.36	24.11 27.09	17.34 23.06	19.04 22.31	13.61 20.88
Insomnia						
*M* *SD*	45.39 35.04	38.29 34.03	35.46 35.71	31.29 29.19	31.97 30.39	23.81 29.65
MFI						
General Fatigue						
*M* *SD*	9.83 4.05	9.00 3.31	9.65 4.11	8.91 3.38	8.68 3.82	8.18 3.64
Physical Fatigue						
*M* *SD*	9.05 3.65	7.80 3.01	8.33 3.56	7.52 3.25	6.61 2.88	6.55 2.93
Reduced Activity						
*M* *SD*	8.60 3.86	8.03 3.57	8.68 4.18	7.84 3.21	7.05 2.97	6.75 2.61
Reduced Motivation						
*M* *SD*	7.23 2.53	6.95 2.74	7.30 2.98	6.52 2.11	6.32 2.36	5.86 2.01
Mental Fatigue						
*M* *SD*	8.38 3.76	7.95 3.45	8.40 4.45	7.98 4.06	7.45 4.31	7.07 3.65

In the explorative univariate analyses, a significant change over time for physical function (*p* = 0.007, η^2^ = 0.052, Fig. 3B), role function (*p* = 0.003, η^2^ = 0.062) and social function (*p* = 0.007, η^2^ = 0.051) was found. The group effect was significant for global health status with higher values in RT (*p* = 0.042, η^2^ = 0.046). Furthermore, the development of AT and RT over time was different for global health status (*p* = 0.034, η^2^ = 0.035).

Planned within-subjects contrasts for t1—t2 showed a significant change over time for role function only (*p* = 0.045, η^2^ = 0.042). Development of AT and RT was different between t1 and t2 for global health status (*p* = 0.027, η^2^ = 0.051) with more sustainable effects in RT (Fig. 3A), and in emotional function (*p* = 0.021, η^2^ = 0.056) with more sustainable effects in RT.

The analyses of the symptom scales of the EORTC-QLQ-C30 for the pooled groups showed no group effect, *F*(3, 92) = 1.946, *p* = 0.128, η^2^ = 0.060, no time effect, *F*(6, 89) = 1.889, *p* = 0.091, η^2^ = 0.113, and no group x time interaction effect, *F*(6, 89) = 2.015, *p* = 0.072, η^2^ = 0.120.

Regarding the univariate explorative analyses, the groups did not differ in any subscale (*p* ≥ 0.057). A significant alteration over time was found for insomnia (*p* = 0.020, η^2^ = 0.041). All other effects failed to reach significance (*p* ≥ 0.055). Planned within subjects-contrasts (t1—t2) revealed a significant decrease over time for insomnia (*p* = 0.047, η^2^ = 0.041) in both groups. Development of pain was different for AT and RT between t1 and t2 (*p* = 0.031, η^2^ = 0.049) with an increase in AT and a decrease in RT.

For the MFI-20, no between-group differences for AT and RT were found, *F*(5, 78) = 1.776, *p* = 0.128, η^2^ = 0.102. Fatigue values did not change over time, *F*(10, 73) = 1.316, *p* = 0.239, η^2^ = 0.153, and the analyses revealed no differences in the development over time between groups, *F*(10, 73) = 0.723, *p* = 0.700, η^2^ = 0.090.

In the univariate analyses, AT and RT differed in physical fatigue (*p* = 0.017, η^2^ = 0.067, Fig. 3E) and reduced motivation (*p* = 0.035, η^2^ = 0.053) with AT showing higher symptom burden. Differences over time were found in physical fatigue (*p* = 0.001, η^2^ = 0.083). For the planned contrasts (t1 – t2) a significant interaction could be obtained only in general fatigue with the RT group decreasing and the AT group increasing (*p* = 0.041, η^2^ = 0.050).

## Discussion

The present study for the first time compared the effects of aerobic and resistance training as well as different work rate-/volume-matched exercise intensity prescriptions on PROs in cancer survivors after primary therapy. Over the intervention period, significant improvements in HRQoL (global health status, function scales physical, role and social function, as well as symptom scale fatigue) were observed. However, the changes over time were not significantly different between groups for global health status and the function scales. Only the symptom scale fatigue showed an increase in AT_Standard_ compared to decreases in all other groups. Similarly, significant improvements in CRF (physical fatigue) were observed, but there were no significant differences in the development over time between groups. Sustainability analyses over a 12-week follow-up period without any further exercise prescription revealed higher sustainability in RT compared to AT for global health status, emotional function and pain. A similar pattern was observed for general fatigue. Furthermore, the significant effects reported in the previous chapter do not necessarily imply a clinically relevant change. Please note that this is a common problem with questionnaire-based studies in medicine.

### Intervention period

The findings of the present study are consistent with the existing body of literature, showing that supervised AT or RT can lead to an improvement in HRQoL [3, 13–18]. However, there are also studies lacking significant effects for some subscales [17, 19]. This heterogeneity might be attributable to different study settings or patient characteristics, in a sense that e.g. group-based workouts might affect role and social functioning more than individual workouts. Surprisingly, we observed an increase in the EORTC-QLQ-C30 symptom scale fatigue in the AT_Standard_ group compared to expected decreases in all other groups, which was indicated by a significant group x time interaction. This deterioration of the AT_Standard_ group is hard to explain. It is inconsistent with the MFI-20 scale general fatigue which showed no significant group x time interaction. Previous studies found significant reductions in fatigue due to training regimens similar to AT_Standard_, as described above. Furthermore, there are no obvious arguments for our observation. Therefore, this might be an incidental finding without any further relevance.

CRF is multidimensional including general, physical and psychosocial domains [20]. Regarding general CRF, no improvements were observed. Contrary to our findings, recent studies and reviews reported improvements in general CRF through AT and RT [3, 15, 21, 22]. In contrast to general CRF, physical fatigue significantly decreased in all training groups. Despite not being significant, a positive trend could be observed for the reduced activity subscale in three training groups (AT_Standard_, AT_Polarized_, RT_Undulating_). Only for the RT_Standard_, which showed the lowest baseline values, no improvement could be observed. This is consistent with recent studies that reported positive effects of RT and AT on physical CRF [15, 23]. For the subscales reduced motivation and mental fatigue, no significant reduction was found. Van Vulpen et al. [24] concluded in their meta-analysis that not all domains of CRF benefit equally from physical exercise. The authors found a positive influence only for general and physical fatigue, but not for the social and affective dimensions [24].

Little is known about the effects of various intensity prescriptions on CRF, especially about polarized AT and daily undulating RT. Taaffe et al. [22] compared the effects of high impact loading (jumping, hopping) plus RT with combined RT and AT in prostate cancer patients undergoing androgen deprivation therapy. All exercise modalities led to improvements in CRF, but in line with our findings, no difference in the effectiveness was found [22]. Mijwel et al. [6] showed positive effects of polarized AT on CRF in breast cancer patients during chemotherapy, which is also in line with our findings. Our results as well as the small amount of available literature indicate that different exercise modes and intensity prescriptions are effective to reduce physical CRF.

Overall, we found no differences in the effects on HRQoL and CRF between the four training interventions. This indicates that aerobic as well as resistance training and “standard” as well as polarized/daily undulating exercise intensity prescriptions can be used to address HRQoL and CRF in cancer survivors after the end of primary therapy. So far, the high AT exercise intensities used for HIIT in the AT_Polarized_ group of the present study are not included in exercise recommendations for cancer survivors [3]. Similarly, the high RT exercise intensities of 4 repetitions at 90% 1RM used in the RT_Undulating_ group are not included in the recommendations [3]. Other current research has also demonstrated that AT_Polarized_ and RT_Undulating_ can be safely performed [11, 25]. Therefore, it might be worth extending the standard recommendation of moderate-to-vigorous-intensity aerobic and resistance training to allow for effective alternatives.

### Sustainability of the training effects

Previous studies and reviews showed favorable follow-up effects of various physical exercise (AT, RT, combined training) on HRQoL and CRF [21, 25–27]. However, little is known about possible differences in the sustainability of training effects between exercise modes. Therefore, we performed follow-up analyses with pooled AT and RT groups. At baseline, the participants of RT showed descriptive higher functionality and lower symptom burden compared to AT. Over the intervention period, global health status descriptively showed improvements in both groups. However, following the intervention, the RT group continued to improve their scores, while the AT group experienced stagnation or deterioration in most subscales except for social function and insomnia symptom burden (see Fig. 2). This is particularly evident in the global health status scale of the EORTC-QLQ-C30, where the difference at follow-up amounted to 10 scale points. These findings are consistent with the results published by Segal et al. [28]. These findings indicate that RT might be superior to AT in situations where sustainability of the training effects on HRQoL plays a major role. This is the case e.g. when cancer patients are facing training interruptions due to treatments like surgeries.

Regarding CRF, the findings for the MFI-20 subscales are in line with those of the EORTC-QLQ-C30 fatigue symptom scale. There was a clear descriptive trend towards further improvements in all CRF domains in the RT group during follow-up which was even significant for the reduced activity subscale. In contrast, the AT group depicted a deterioration of all subscales during follow-up. This suggests that RT might be superior to AT with regard to sustainable effects on CRF. Similar results were reported by Segal et al. [28].

There are strengths and weaknesses of the present study that should be considered. To our knowledge, this is the first study that compared the effects of different intensity prescriptions of matched work rate for AT and matched volume for RT. RT_Undulating_ and AT_Polarized_ followed rare prescriptions and more studies are needed to verify the present findings. However, a better reporting of all FITT-criteria is crucial to improve comparability of exercise oncology trials [29, 30]. By including breast and prostate cancer survivors, the most common cancer types worldwide were covered [31]. Although more than 120 participants are a large sample, the study was powered for the primary endpoint VO_2peak_. Therefore, significant effects on PROs might have been missed. In this study, participants performed two sessions/week. It is possible that for some individuals a higher volume would have let to different outcomes. However, the ability to tolerate exercise may differ between cancer survivors [3]. Furthermore, we only broadly recorded the exercise behavior of the participants over the 12-week follow-up period. Therefore, no conclusion can be drawn on whether the difference in the sustainability of the training effects is attributable to the exercise mode or differences in the physical activity levels in the follow-up period. Future studies should report the physical activity levels and training regimes of their participants after intervention, e.g. by means of activity trackers. And finally, a socioeconomic bias cannot be excluded because group allocation was based on the available equipment in the local training facilities nearby patients' homes.  

## Perspective

The present four-arm training intervention trial investigated the effects of AT and RT as well as different work rate-/volume-matched exercise intensities on PROs in breast and prostate cancer patients after the end of primary therapy. A significant favourable effect was observed over the intervention period for HRQoL and CRF. However, no training regimen was superior to another. Therewith, polarized AT and daily undulating RT appear to be adequate alternatives to the moderate-to-high intensity AT and RT prescriptions given in the current exercise recommendations for cancer survivors [3]. Analysis of a 12-week follow-up period without any further exercise prescription indicates that RT might elicit more sustainable effects on HRQoL and CRF. However, these partly non-significant findings need to be interpreted with caution and further studies are needed to confirm them.

## Data Availability

Data are available upon request from the corresponding author.
